# Preeclampsia With Posterior Reversible Encephalopathy Syndrome at 19 Weeks Gestation Resulting in Intrauterine Fetal Demise

**DOI:** 10.7759/cureus.55490

**Published:** 2024-03-04

**Authors:** Zara H Siddiqui, Kyle A Cohen, Jonathan Scott

**Affiliations:** 1 Obstetrics and Gynecology, Alabama College of Osteopathic Medicine, Dothan, USA; 2 Obstetrics and Gynecology, Women's Medical Center, Dothan, USA

**Keywords:** preeclampsia complications, fetal loss, hypertensive disorders in pregnancy (hdp), intrauterine fetal demise, early-onset preeclampsia, preeclampsia with severe features, posterior reversible encephalopathy syndrome (pres)

## Abstract

Posterior reversible encephalopathy syndrome (PRES) can be defined as a clinical syndrome of headache, seizures, visual disturbance, altered mental status, and characteristic magnetic resonance imaging (MRI) findings of vasogenic edema in the posterior subcortical parietal-occipital white matter. There are numerous potential inciting factors, including immunosuppression, renal disease, malignancy, cytotoxic medications, hypertension, preeclampsia, and eclampsia. In this paper, we present the case of a 21-year-old female at 19 weeks gestation presenting with symptoms consistent with preeclampsia with severe features and PRES. She was transferred to our facility after initial stabilization. She had an atypical course of preeclampsia prior to 20 weeks gestation, PRES lacking seizure activity, and ultimately her case resulted in intrauterine fetal demise (IUFD) at 20 weeks and six days gestation. As indicated by its name, PRES is considered a fully reversible syndrome, and the patient recovered after stabilization of her hypertensive disorder and delivery of the fetus. This case illustrates the importance of prompt recognition and treatment of hypertensive disorders in pregnant patients and the possibility of complications that can result in significant morbidity and mortality for both the mother and fetus.

## Introduction

Preeclampsia is defined as the new onset of hypertension and proteinuria or hypertension with evidence of end-organ dysfunction with or without proteinuria in a previously normotensive patient, typically after 20 weeks of gestation or in the postpartum period [1]. The American College of Obstetrics and Gynecology (ACOG) defines the hypertension component of preeclampsia as a systolic blood pressure (SBP) of 140 mmHg or higher and/or a diastolic blood pressure (DBP) of 90 mmHg or higher on two occasions at least four hours apart [1]. The proteinuria component of preeclampsia is defined as a 24-hour urine collection with 300 mg or more of protein, a protein/creatinine ratio of 0.3 mg/dL or more, or a urine dipstick reading of 2+. For the pregnant patient with hypertension and no proteinuria, preeclampsia can also be defined by signs of end-organ failure, such as thrombocytopenia, renal insufficiency with serum creatinine greater than 1.1 mg/dL, doubling serum creatinine without preexisting renal disease, liver impairment with elevated liver transaminases to greater than two times the normal range, pulmonary edema, or new onset headache refractory to medication [1]. Preeclampsia with severe features is defined as hypertension with SBP of 160 mmHg or higher and/or DBP of 110 mmHg or higher and alarm symptoms of persistent or severe headache, visual disturbances, upper abdominal or epigastric pain, altered mental status, or new dyspnea or orthopnea [1]. Rare or atypical cases of preeclampsia include preeclampsia prior to 20 weeks, onset or exacerbation of symptoms greater than two days postpartum, severe features without hypertension, isolated hypertension, and isolated proteinuria. Other conditions associated with preeclampsia include hemolysis, elevated liver enzymes, and low platelets (HELLP syndrome) and posterior reversible encephalopathy syndrome (PRES).

PRES is a rare neurological disorder that was first identified by Hinchley et al. in 1996 as a posterior reversible leukoencephalopathy syndrome, which is mainly a clinical-radiological diagnosis [2]. Diagnosis is typically made based on a constellation of symptoms that are supported by risks of disease and radiologic studies [3]. General symptoms include visual disturbance, headache, altered mental status, seizures, or nausea and vomiting. Risks and associated preexisting diseases include hypertension, preeclampsia, eclampsia, renal dysfunction, cytotoxic and immunosuppressive medications, solid organ and bone marrow transplantation, autoimmune disorders, certain toxins, sepsis, and malignancy [4]. Interestingly, PRES is associated with approximately 98% of cases of eclampsia [4]. The syndrome may present at any age, but on average, it presents in young to middle age and more often affects females than males [4].

The diagnosis is supported by radiological findings utilizing either computed tomography (CT) or magnetic resonance imaging (MRI). Bartynski and Boardman identified the most common PRES-related neuroimaging patterns on MRI to be vasogenic edema of the parietal or occipital lobes (98%), frontal lobes (68%), inferior temporal lobes (40%), and cerebellar hemispheres (30%) [5]. In addition, three main patterns of neuroimaging have been noted in PRES, including the holo-hemispheric watershed, superior frontal sulcus, and dominant parietal-occipital lobes [5]. Another pattern in neuroimaging that has been identified involves the brainstem, basal ganglia, posterior limb of the internal capsule, cerebellum, and periventricular regions [6].

The following report illustrates the case of a pregnant female at 19 weeks gestation presenting with preeclampsia with severe features and PRES, which ultimately resulted in intrauterine fetal demise (IUFD) but full recovery of the patient.

## Case presentation

A 21-year-old G2P1001 female presented to an outside facility with a chief complaint of headache for two days. She reported that her headache was 10/10 in severity. She also reported blurry vision and confusion. Her partner at the bedside reported that she had increased confusion for the past day. The patient recently learned of her pregnancy one week prior and had not yet sought prenatal care. She was unsure of the date of her last menstrual period and was therefore unaware of the current gestational age of the pregnancy. They reported that a cold had been going through their household for the past week, but otherwise, the patient had been feeling well prior to the onset of the headache. The patient reported no past medical or surgical history. She denied the use of tobacco products, alcohol, or illicit drugs. The patient and her partner have a two-year-old healthy son.

On admission to the outside facility, her vital signs were blood pressure of 220/126, temperature of 99.2° F, heart rate of 91 beats per minute (bpm), respiratory rate of 22, and O_2_ saturation of 96% on room air. In the emergency department, she was started on IV (intravenous) nicardipine and admitted to the intensive care unit (ICU). Initial labs were significant for an elevated white blood cell count (Table 1). Urinalysis revealed 3+ protein, 2+ ketones, 1+ bilirubin, and 3+ blood and was negative for leukocytes or nitrites. The urine drug screen was negative. The respiratory viral panel was positive for parainfluenza virus and negative for COVID-19. Beta-hCG was 24,588 mIU/mL. Blood and urine cultures were obtained.

**Table 1:** Initial patient lab values WBC: white blood cells, PT: prothrombin time, PTT: partial thromboplastin time, INR: international normalized ratio, BUN: blood urea nitrogen

Lab	Patient’s value	Reference range
WBC	17,200	4500-11,000/mL
Platelets	184,000	150,000-450,000/mL
Hemoglobin	11.8	12-16 g/dL (female)
Hematocrit	38.5	37-47% (female)
Sodium	133	136-145 mEq/L
Potassium	3.5	3.5-5.0 mEq/L
Magnesium	1.7	1.6-2.6 mg/dL
Lactate	2.2	0.7-2.1 mmol/L
Glucose	117	<140 mg/dL
PT	9.7	11-13 seconds
PTT	33.1	25-35 seconds
INR	0.86	<1.1
Creatinine	1.06	0.5-1.10 mg/dL (female)
BUN	13	8-20 mg/dL
Albumin	2.9	3.5-5.5 g/dL
Urinalysis	3+ protein, 2+ ketones, 1+ bilirubin, 3+ blood	negative
Respiratory viral panel	+PIV	negative
Beta-hCG	24,588	variable

After evaluation and stabilization, the patient was sent for a CT of the head, which revealed cortical edema involving the posterior and occipital regions of the brain. She was subsequently started on 20 mg IV labetalol, 1000 mg oral acetaminophen, 4 mg IV magnesium, and 1 g IV Rocephin, and the nicardipine drip was continued. At this point, the patient was transferred to our facility for a higher level of care and obstetrics evaluation.

Upon admission to our facility, the patient’s partner reported that the patient seemed increasingly confused. Vital signs were blood pressure of 150/98, pulse of 96 bpm, temperature of 99°F, respiratory rate of 18, and O_2_ saturation of 100% on room air. Physical examination revealed the patient in acute distress and ill-appearing. Extraocular movements were intact but with some difficulty in tracking. Cardiovascular examination revealed the heart with a regular rate and rhythm. Respiratory effort was normal and lungs were clear to auscultation. An abdominal exam revealed normal bowel sounds and a soft abdomen without tenderness, distension, masses, rebound, or guarding. Upon neurological examination, the patient was alert and was able to follow commands but oriented only to a person and unable to answer some questions. No cranial nerve or sensory deficits were noted. She had 5/5 upper and lower extremity strength bilaterally. Coordination tests revealed a normal finger-nose-finger test but with a delayed response. She displayed delayed rapid alternating movements. There were no signs of seizure-like activity.

Obstetrics evaluation was obtained, and a transvaginal ultrasound was ordered, which revealed an intrauterine fetus in variable presentation with a gestational age of 19 weeks and one day. The fetal heart rate was 144 bpm. The placenta was in a posterior position with no evidence of previa. Additional laboratory studies obtained at our facility revealed a urine protein of >1000 mg/dL and a protein-to-creatinine ratio of >5662 mg/g. Repeat urinalysis revealed 3+ protein, 1+ blood, and trace ketones. The 24-hour urine revealed protein >12,500 mg. She tested positive for rubella antibodies. She tested negative for human immunodeficiency virus (HIV) and hepatitis B. Antinuclear antibodies (ANA), anti-double-stranded deoxyribonucleic acid (dsDNA) antibodies, glomerular basement membrane antibodies, beta-2 glycoprotein I antibodies, and cardiolipin antibodies were negative. Urine metanephrines, vanillylmandelic acid (VMA), and catecholamines were within normal limits. Urine cultures and two peripheral blood cultures repeatedly showed no growth.

**Figure 1:**
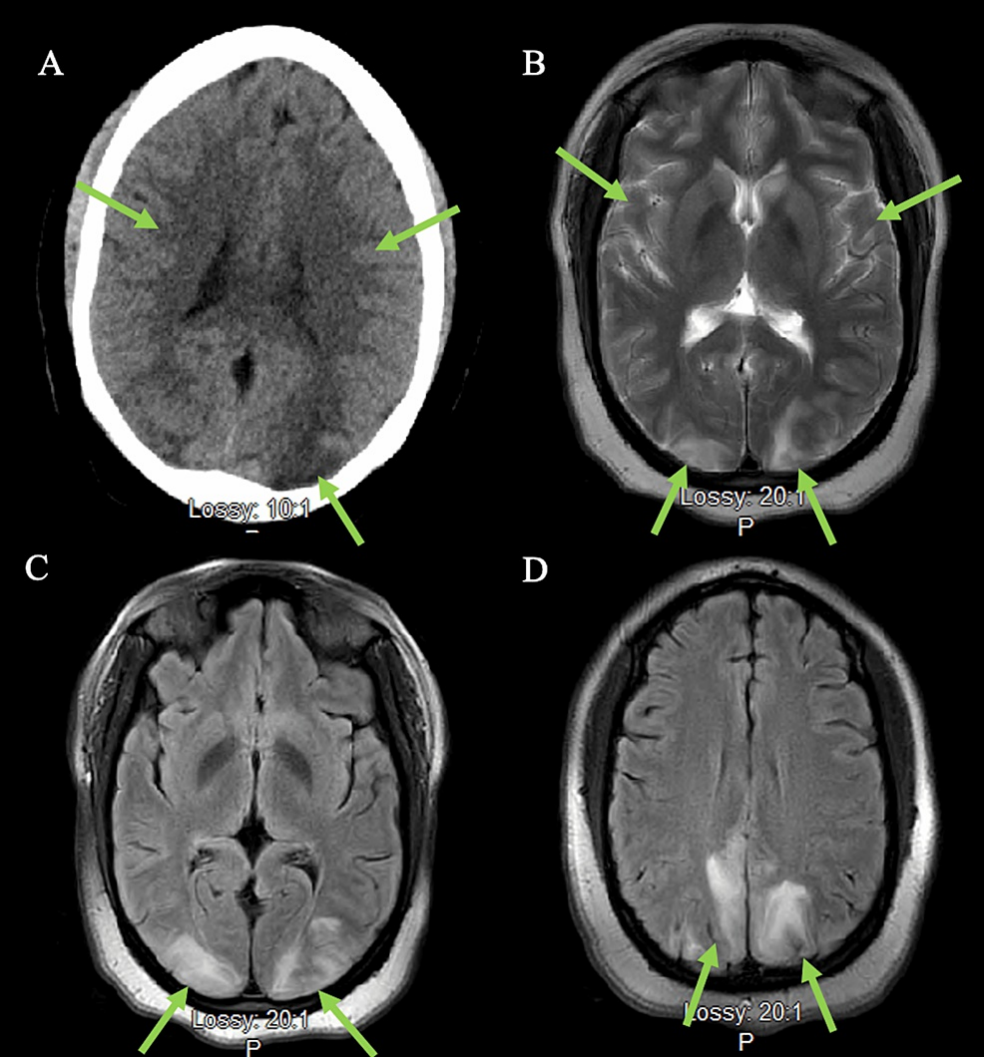
Neuroimaging findings A: Non-contrast head CT demonstrating vasogenic edema of the parieto-occipital lobes (see arrows). B: T2 MRI showing cortical and subcortical edema of bilateral parietal and occipital lobes (see arrows). C, D: Fluid attenuated inversion recovery (FLAIR) MRI demonstrating cortical edema centered on the occipital lobes and inferior margin of the parietal lobes (see arrows).

CT of the head without contrast revealed bilateral vasogenic edema of the parieto-occipital brain parenchyma and a portion of the left anterior frontal lobe (Figure 1A). No hydrocephalus, hemorrhage, or herniation was noted. MRI of the head revealed symmetric and patchy areas of the cortical and subcortical edema of the bilateral parietal and occipital lobes, posterior corpus callosum, and bilateral caudate nuclei (Figure 1B, 1C, 1D). MR venography (MRV) of the head revealed a normal flow-related signal in the intracranial venous sinuses with no evidence of venous sinus thrombosis. An electroencephalogram (EEG) was conducted and revealed mildly slow background activity with no epileptiform abnormalities, suggesting non-specific encephalopathy.

On day two of admission, the patient’s blood pressure improved with continuation of antihypertensive medication. WBC decreased to 14,000/mL. Fetal monitoring continued to be reassuring. Due to the risks for complications and the severity of the patient’s condition, she was transferred from our facility to a nearby tertiary care center with maternal-fetal medicine service. The patient subsequently experienced IUFD at 20 weeks and six days gestation. Placental weight at delivery was 46 grams (normal expected range of 136-140 grams at this gestational age).

## Discussion

This report illustrates a rare case of preeclampsia at 19 weeks gestation. Given the patient’s severe hypertension, 3+ proteinuria, protein-to-creatinine ratio >5000 mg/g, 24-hour urine protein >12,500 mg, severe headache, confusion, and visual disturbance, she was diagnosed with preeclampsia with severe features. The patient had no known risk factors for preeclampsia, including nulliparity, prior history of hypertension, age >35, prior preeclampsia, diabetes or gestational diabetes, antiphospholipid antibody syndrome, molar pregnancy, or renal or liver disease. The patient was also diagnosed with PRES given her clinical syndrome of headaches, altered mental status, visual disturbance, nausea, and classic neuroimaging findings on MRI.

In a 2023 systematic review of atypical preeclampsia prior to 20 weeks gestation, 37 total cases were identified worldwide, ranging from onset at 14 weeks to 19 weeks gestation [7]. Of these cases, five resulted in live births, nine had IUFD, and 23 had terminations of pregnancy [7]. A majority of these patients had preexisting risk factors, such as antiphospholipid antibody syndrome, protein S deficiency, and pre-pregnancy hypertension; our patient lacked these risk factors [7]. Another case study conducted in 2014 reported a patient presenting with features of hypertension and proteinuria at 18 weeks gestation that also resulted in IUFD; this author argued the presence of hypertension and proteinuria prior to 20 weeks suggests underlying renal disease presenting as a nephrotic syndrome [8]. Our patient did not have a renal biopsy, but the lack of preexisting renal disease ruled this possibility out at the time. None of the patients in the case studies mentioned the presence of PRES or seizure activity. Given the rare occurrence of preeclampsia prior to 20 weeks gestation, it is important that clinicians be aware of its possibility and symptoms or high-risk features to look for to avoid morbidity and mortality.

Another unique feature of this patient case is the occurrence of PRES with atypical features, including the lack of seizure activity, and resulting IUFD. Some case reports have been published discussing IUFD as a result of PRES. For example, Yeh et al. describe a case of PRES in a 35-year-old Asian woman two days following IUFD and subsequent cesarean delivery of a twin pregnancy at 32 weeks gestation; this patient had no signs of hypertension or proteinuria but did have symptoms, including focal seizures and visual disturbance along with classic neuroimaging findings [9]. Another report presents the case of a 20-year-old woman in the third trimester of pregnancy who was found unconscious and in early labor and with a fetus with no heartbeat; she had signs and symptoms consistent with HELLP (hemolysis, elevated liver enzymes, and low platelets), severe hypertension, and neuroimaging findings consistent with PRES; a cesarean section was performed leading to the delivery of a stillborn fetus [10].

Another case reports a 27-year-old African American patient with neuromyelitis optica in pregnancy presenting at 31 weeks gestation with signs of HELLP syndrome, eclamptic seizures, neuroimaging findings consistent with PRES, and subsequent IUFD, which occurred during the hospitalization and stabilization of the patient [11].

IUFD as a result of PRES is an uncommon finding, and there are many factors that can be hypothesized to contribute. The causes may differ based on the presence or absence of preeclampsia, eclampsia, seizure activity, or other risk factors. In our patient presenting with preeclampsia with severe features along with a small placenta but without seizure activity, it can be hypothesized that the maladaptive remodeling of the placenta led to placental malperfusion and subsequent fetal hypoxia, which contributed to the fetal demise. The pathophysiology of preeclampsia is related to defective spiral artery remodeling early on in pregnancy leading to uteroplacental malperfusion [12]. This imposes stress on the syncytiotrophoblast cells (the epithelial cells that form the placental barrier between maternal and fetal blood), which leads to a release of factors, including anti-angiogenic factors, pro-inflammatory cytokines, and cell-free fetal DNA [12]. These factors when released into the maternal bloodstream impair maternal endothelial function, invoking a systemic inflammatory response resulting in the clinical symptoms of preeclampsia [12]. As a result of chronic uteroplacental ischemia, there is impaired blood flow to the fetus, leading to complications, including oligohydramnios, preterm birth, fetal growth restriction, placental abruption, and fetal demise [1]. The low placental weight for this gestational age (46 grams) in the case report is consistent with preeclampsia and uteroplacental ischemia.

In addition, PRES typically presents with a clinical syndrome of constant generalized headaches, visual disturbance, altered consciousness, and seizure activity. Our patient did not present with seizure activity, which is atypical and only occurs in a minority of patients with PRES [13]. One retrospective clinical series found seizures as a presenting symptom in 87% of patients, encephalopathy in 92%, visual symptoms in 39%, and headaches in 53% [14].

The theory surrounding the pathogenesis of PRES involves various mechanisms. One of these theories is that rapid increases in blood pressure lead to a failure of autoregulation of blood vessels in which arterioles dilate leading to increased cerebral blood flow with rises in systemic blood pressure; this leads to hyperperfusion of the brain and a breakdown of the blood-brain barrier, which allows for extravasation of fluid and blood products into the brain parenchyma [15]. Another theory also involves autoregulatory failure of blood vessels, but states that there is reactive focal vasoconstriction or vasospasm in the brain that leads to ischemia and cerebral infarction, which could also be a result of cerebral edema causing compression of the microcirculation [16,17]. Preeclampsia is a known risk factor for PRES and likely involves endothelial dysfunction as a result of the anti-angiogenic and pro-inflammatory factors released from the placenta as a result of dysfunctional spiral artery remodeling. It is possible that endothelial dysfunction in PRES is a result of the abnormal implantation of the fetus, poor placental perfusion, or the overall poorly perfused fetoplacental unit secreting cytotoxic factors that exist in the theory of the mechanism of preeclampsia [18].

The management of preeclampsia and PRES are similar and involve the reduction of blood pressure and early delivery of the fetus. For patients with preeclampsia, delivery is recommended at 37 weeks even if the patient is asymptomatic [19]. For those with severe complications and risk of maternal mortality, delivery is recommended regardless of gestational age [19]. In addition, treatment with antihypertensive therapy with labetalol, nifedipine, or hydralazine is warranted [19]. For patients with PRES, additional therapy may include seizure prophylaxis, seizure management, discontinuing causative agents, treating underlying conditions, and delivery of the fetus and placenta. There is also a recommendation for aspirin therapy for the prevention of preeclampsia in patients with high-risk features, including previous preeclampsia, multi-fetal gestation, age >35, BMI >30, renal disease, autoimmune disease, diabetes, and chronic hypertension [1]. The prognosis for PRES is excellent with most cases resolving completely after delivery. One retrospective clinical series found that symptoms were reversible and resolved in a mean of 5.3 days [14].

## Conclusions

This patient case included unique features, including preeclampsia prior to 20 weeks gestation, no known patient risk factors for preeclampsia or PRES, PRES without seizure activity, and preeclampsia/PRES resulting in IUFD. It is likely that the patient’s history of preeclampsia with severe hypertension and other severe features triggered the development of PRES. The patient was treated appropriately with antihypertensive medication and seizure prophylaxis, but the patient still suffered loss of the fetus. This case emphasizes the importance of clinicians to be cognizant of the risk factors for preeclampsia, but also of the fact that patients may present with early new onset preeclampsia without any warning signs. It is important for obstetricians to be aware of the possibility of PRES and to be able to enact rapid diagnosis via MRI and rapid management to avoid serious complications. Given the patient’s rapid onset of symptoms and lack of prenatal care, this particular outcome may not have been avoidable. However, it stresses the importance of appropriate early prenatal care with careful monitoring of blood pressure, routine screening for proteinuria, and all other potential risk factors for the development of preeclampsia. Suggestions for further research include solidifying the epidemiologic and physiologic relationship between preeclampsia and PRES, elucidating potential causes of preeclampsia in patients with no known risk factors, and further investigating the pathophysiology of PRES.
